# Supplementary material for: Effect of hypoxia conditioning on physical fitness in middle-aged and older adults—a systematic review and meta-analysis
Source: PeerJ. 2025 May 23;13:e19348. doi: 10.7717/peerj.19348 (PMC12105624; doi:10.7717/peerj.19348)

**PUBMED 18 RCT**

(((((Middle Aged[MeSH Terms]) OR (Middle Age[Title/Abstract])) OR ((Aged[MeSH Terms]) OR (Elderly[Title/Abstract]))) AND (((((((((((((((((((((((((((((Exercise[MeSH Terms]) OR (Exercises[Title/Abstract])) OR (Physical Activity[Title/Abstract])) OR (Activities, Physical[Title/Abstract])) OR (Activity, Physical[Title/Abstract])) OR (Physical Activities[Title/Abstract])) OR (Exercise, Physical[Title/Abstract])) OR (Exercises, Physical[Title/Abstract])) OR (Physical Exercise[Title/Abstract])) OR (Physical Exercises[Title/Abstract])) OR (Acute Exercise[Title/Abstract])) OR (Acute Exercises[Title/Abstract])) OR (Exercise, Acute[Title/Abstract])) OR (Exercises, Acute[Title/Abstract])) OR (Exercise, Isometric[Title/Abstract])) OR (Exercises, Isometric[Title/Abstract])) OR (Isometric Exercises[Title/Abstract])) OR (Isometric Exercise[Title/Abstract])) OR (Exercise, Aerobic[Title/Abstract])) OR (Aerobic Exercise[Title/Abstract])) OR (Aerobic Exercises[Title/Abstract])) OR (Exercises, Aerobic[Title/Abstract])) OR (Exercise Training[Title/Abstract])) OR (Exercise Trainings[Title/Abstract])) OR (Training, Exercise[Title/Abstract])) OR (Trainings, Exercise[Title/Abstract])) OR (((((((((((((High-Intensity Interval Training[MeSH Terms]) OR (High Intensity Interval Training[Title/Abstract])) OR (High-Intensity Interval Trainings[Title/Abstract])) OR (Interval Training, High-Intensity[Title/Abstract])) OR (Interval Trainings, High-Intensity[Title/Abstract])) OR (Training, High-Intensity Interval[Title/Abstract])) OR (Trainings, High-Intensity Interval[Title/Abstract])) OR (High-Intensity Intermittent Exercise[Title/Abstract])) OR (Exercise, High-Intensity Intermittent[Title/Abstract])) OR (Exercises, High-Intensity Intermittent[Title/Abstract])) OR (High-Intensity Intermittent Exercises[Title/Abstract])) OR (Sprint Interval Training[Title/Abstract])) OR (Sprint Interval Trainings[Title/Abstract]))) OR ((((((((((((((((((((((((Resistance Training[MeSH Terms]) OR (Training, Resistance[Title/Abstract])) OR (Strength Training[Title/Abstract])) OR (Training, Strength[Title/Abstract])) OR (Weight-Lifting Strengthening Program[Title/Abstract])) OR (Strengthening Program, Weight-Lifting[Title/Abstract])) OR (Strengthening Programs, Weight-Lifting[Title/Abstract])) OR (Weight Lifting Strengthening Program[Title/Abstract])) OR (Weight-Lifting Strengthening Programs[Title/Abstract])) OR (Weight-Lifting Exercise Program[Title/Abstract])) OR (Exercise Program, Weight-Lifting[Title/Abstract])) OR (Exercise Programs, Weight-Lifting[Title/Abstract])) OR (Weight Lifting Exercise Program[Title/Abstract])) OR (Weight-Lifting Exercise Programs[Title/Abstract])) OR (Weight-Bearing Strengthening Program[Title/Abstract])) OR (Strengthening Program, Weight-Bearing[Title/Abstract])) OR (Strengthening Programs, Weight-Bearing[Title/Abstract])) OR (Weight Bearing Strengthening Program[Title/Abstract])) OR (Weight-Bearing Strengthening Programs[Title/Abstract])) OR (Weight-Bearing Exercise Program[Title/Abstract])) OR (Exercise Program, Weight-Bearing[Title/Abstract])) OR (Exercise Programs, Weight-Bearing[Title/Abstract])) OR (Weight Bearing Exercise Program[Title/Abstract])) OR (Weight-Bearing Exercise Programs[Title/Abstract]))) OR ((strength training[Title/Abstract]) OR (weight training[Title/Abstract])))) AND ((((((((((Hypoxia[MeSH Terms]) OR (Oxygen Deficiency[Title/Abstract])) OR (Deficiency, Oxygen[Title/Abstract])) OR (Deficiencies, Oxygen[Title/Abstract])) OR (Oxygen Deficiencies[Title/Abstract])) OR (Hypoxemia[Title/Abstract])) OR (Anoxia[Title/Abstract])) OR (Anoxemia[Title/Abstract])) OR ((altitude[MeSH Terms]) OR (altitudes[Title/Abstract]))) OR ((normobaric[Title/Abstract]) OR (hypoxic exposure[Title/Abstract])))) AND (((Exercise performance[Title/Abstract]) OR ((((((((((((((Physical Functional Performance[MeSH Terms]) OR (Functional Performance, Physical[Title/Abstract])) OR (Functional Performances, Physical[Title/Abstract])) OR (Performance, Physical Functional[Title/Abstract])) OR (Performances, Physical Functional[Title/Abstract])) OR (Physical Functional Performances[Title/Abstract])) OR (Functional Performance[Title/Abstract])) OR (Functional Performances[Title/Abstract])) OR (Performance, Functional[Title/Abstract])) OR (Performances, Functional[Title/Abstract])) OR (Physical Performance[Title/Abstract])) OR (Performance, Physical[Title/Abstract])) OR (Performances, Physical[Title/Abstract])) OR (Physical Performances[Title/Abstract]))) OR ((Physical Fitness[MeSH Terms]) OR (Fitness, Physical[Title/Abstract])))


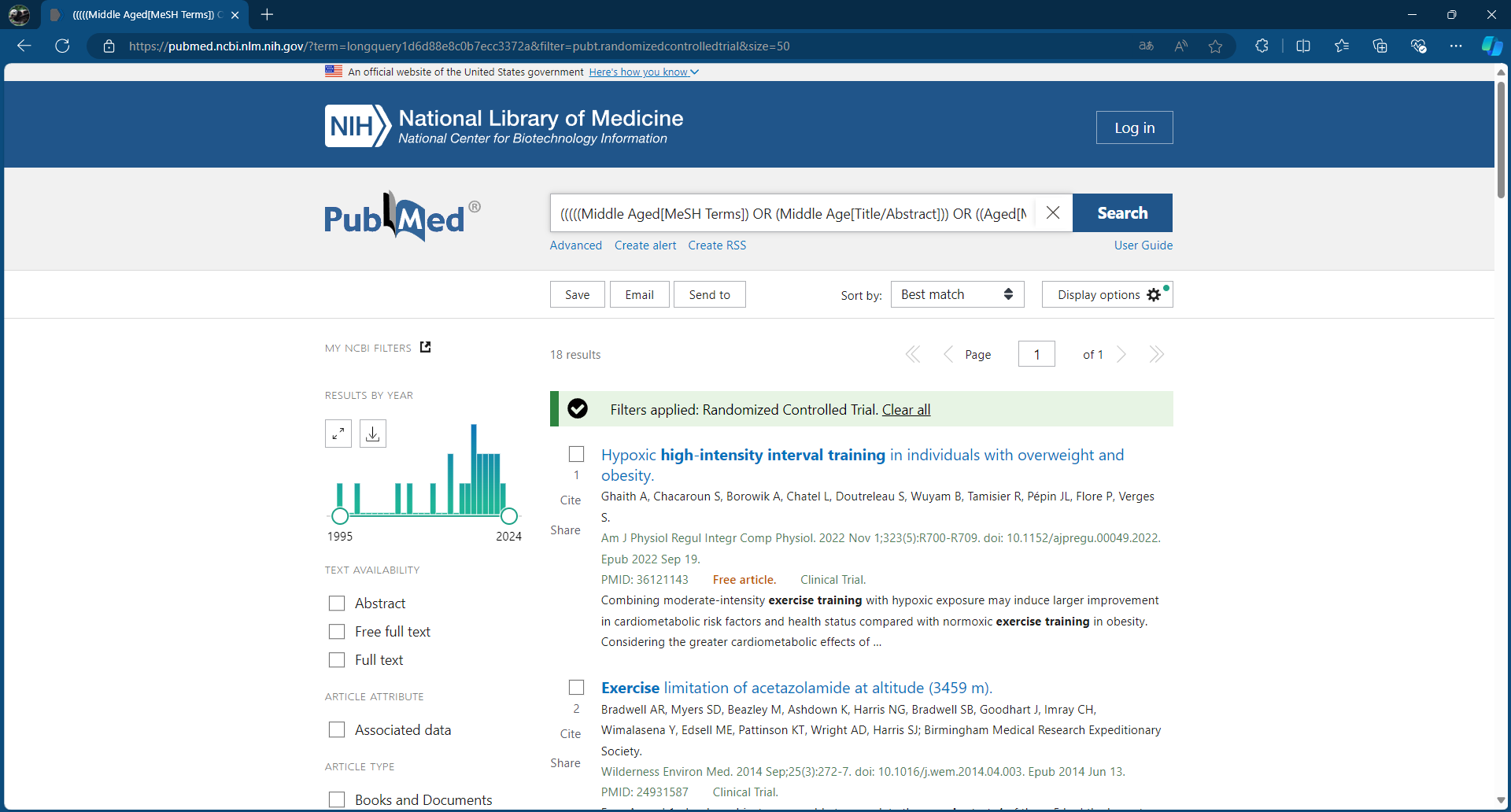


**Embase 41**

('aged'/exp OR 'aged':ti,ab OR 'aged patient':ti,ab OR 'aged people':ti,ab OR 'aged person':ti,ab OR 'aged subject':ti,ab OR 'elderly':ti,ab OR 'elderly patient':ti,ab OR 'elderly people':ti,ab OR 'elderly person':ti,ab OR 'elderly subject':ti,ab OR 'senior citizen':ti,ab OR 'senium':ti,ab OR 'middle aged'/exp OR 'middle age':ti,ab OR 'middle aged':ti,ab) AND ('hypoxia'/exp OR 'deficiency, oxygen':ti,ab OR 'diffusion anoxia':ti,ab OR 'diffusion hypoxia':ti,ab OR 'hypoxia':ti,ab OR 'hypoxia warning':ti,ab OR 'hypoxic drive':ti,ab OR 'hypoxic stress':ti,ab OR 'oxygen deficiency':ti,ab OR 'altitude'/exp OR 'altitude':ti,ab OR 'high altitude':ti,ab OR 'oxygen tension'/exp OR 'normobaric oxygen':ti,ab OR 'oxygen pressure':ti,ab OR 'oxygen tension':ti,ab OR 'oxygen tension recording':ti,ab OR 'p o2':ti,ab OR 'p.o. 2':ti,ab OR 'po2':ti,ab OR 'partial oxygen pressure':ti,ab OR 'partial oxygen tension':ti,ab OR 'po 2':ti,ab OR 'po2 determination':ti,ab OR 'po2 measurement':ti,ab OR 'tension, oxygen':ti,ab OR 'hypoxic exposure':ti,ab) AND ('physical fitness':ti,ab OR 'physical performance'/exp OR 'assessment, physical performance':ti,ab OR 'evaluation, physical performance':ti,ab OR 'performance, physical':ti,ab OR 'physical functional performance':ti,ab OR 'physical performance':ti,ab OR 'physical performance assessment':ti,ab OR 'physical performance evaluation':ti,ab OR 'physical performancy':ti,ab OR 'exercise performance':ti,ab) AND ('randomized controlled trial'/exp OR 'controlled trial, randomized' OR 'randomised controlled study' OR 'randomised controlled trial' OR 'randomized controlled study' OR 'randomized controlled trial' OR 'trial, randomized controlled')

**WOS 128**

(((TS=(Hypoxia)) OR TS=(Altitude)) OR TS=(normobaric)) OR TS=(hypoxic exposure)

((((TS=(Exercise)) OR TS=(High-Intensity Interval Training)) OR TS=(Resistance Training)) OR TS=(strength training)) OR TS=(weight training)

(TS=(Aged)) OR TS=(Middle Aged)

((TS=(Physical Functional Performance)) OR TS=(Physical Fitness)) OR TS=(Exercise performance)

TS=(clinical trial* OR research design OR comparative stud* OR evaluation stud* OR controlled trial* OR follow-up stud* OR prospective stud* OR random* OR placebo* OR single blind* OR double blind*)

**COCHRANE 26**

MeSH descriptor: [Hypoxia] explode all trees

MeSH descriptor: [Altitude] explode all trees

normobaric

hypoxic exposure

MeSH descriptor: [Exercise] explode all trees

MeSH descriptor: [High-Intensity Interval Training] explode all trees

MeSH descriptor: [Resistance Training] explode all trees

strength training

weight training

MeSH descriptor: [Physical Fitness] explode all trees

MeSH descriptor: [Physical Functional Performance] explode all trees

Exercise performance

MeSH descriptor: [Aged] in all MeSH products

MeSH descriptor: [Middle Aged] explode all trees


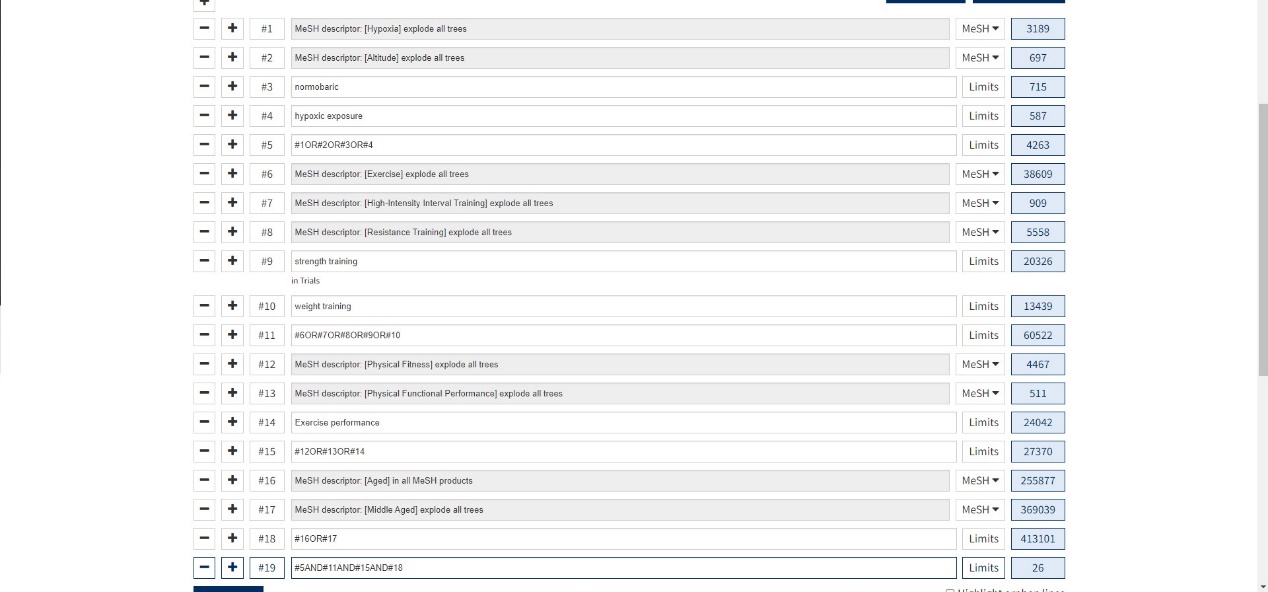


**Quality of evidence**

| Study characteristics | **Functional outcomes** | | **Muscle strength** | | **Power output** | | **VO_2max_** | | **VO_2peak_** | | **Exercise workload** | |
| --- | --- | --- | --- | --- | --- | --- | --- | --- | --- | --- | --- | --- |
|  | N | Effect Size (95%CI) | N | Effect Size (95%CI) | N | Effect Size (95%CI) | N | Effect Size (95%CI) | N | Effect Size (95%CI) | N | Effect Size (95%CI) |
| **Age** |  |  |  |  |  |  |  |  |  |  |  |  |
| middle-aged | - | - | - | - | 4 | 0.35 (-0.40, 1.10)/ L | 4 | 0.90 (-0.68, 2.48)/ VL | 5 | 0.37 (0.03, 0.70)***/** H | 4 | -10.07 (-34.95, 14.80)/ VL |
| older | 3 | -0.21 (-0.66, 0.24)/ M | 3 | -0.19 (-0.63, 0.26)/ M | 2 | 0.26 (-0.21, 0.74)/ H | - | - | - | - | - | - |
| **Obese** |  |  |  |  |  |  |  |  |  |  |  |  |
| yes | - | - | - | - | 3 | 0.35 (-0.09, 0.80)/ L | 4 | 0.90 (-0.68, 2.48)/ VL | 4 | 0.41 (0.04, 0.79)***/** M | 3 | -13.83 (-48.95, 21.30)/ VL |
| no | 2 | -0.13 (-0.67, 0.41)/ M | 2 | -0.39 (-0.93, 0.14)/ M | 3 | 0.15 (-0.24, 0.55)/ H | - | - | 2 | 0.13 (-0.35, 0.62)/ H | - | - |
| **Intervention Duration** |  |  |  |  |  |  |  |  |  |  |  |  |
| ≤ 8 weeks | - | - | 2 | -0.39 (-0.93, 0.14)/ M | 4 | 0.23 (-0.15, 0.61)/ M | 5 | 0.39 (-1.12, 1.90)/ VL | 5 | 0.37 (0.04, 0.70)***/** H | 4 | -10.07 (-34.95, 14.80)/ VL |
| ＞8 weeks | 2 | -0.09 (-0.26, 0.08)/ H | - | - | 2 | 0.26 (-0.21, 0.74)/ H | - | - | - | - | - | - |
| **Hypoxia duration** |  |  |  |  |  |  |  |  |  |  |  |  |
| ≥ 60 min | 2 | -0.28 (-0.79, 0.24)/ M | 3 | -0.19 (-0.63, 0.26)/ M | 4 | 0.14 (-0.20, 0.48)/ H | 3 | 0.52 (-1.43, 2.48)/ VL | 3 | 0.12 (-0.28, 0.52)/ H | 2 | -26.86 (-65.06, 11.34)/ VL |
| ＜60min | - | - | - | - | 2 | 0.78 (-1.01, 2.58)/ L | - | - | 3 | 0.55 (0.10, 0.99)***/** M | 2 | 5.11 (-13.75, 23.98)/ L |
| **FiO2** |  |  |  |  |  |  |  |  |  |  |  |  |
| ≤ 15% | - | - | 2 | -0.51 (-2.76, 1.74)/ L | 4 | 0.35 (-0.40, 1.10)/ M | 5 | 0.39 (-1.12, 1.90)/ VL | 4 | 0.36 (-0.02, 0.74)/ M | 3 | -16.89 (-44.14, 10.36)/ VL |
| ＞15% | - | - | - | - | - | - | - | - | - | - | - | - |
| **Intervention type** |  |  |  |  |  |  |  |  |  |  |  |  |
| Aerobic | - | - | - | - | 4 | 0.41 (-0.27, 1.10)/ L | 3 | 1.06 (-1.28, 3.40)/ VL | 4 | 0.39 (0.01, 0.77) ***/** M | 3 | 0.03 (-14.73, 14.79)/ L |
| Resistance | - | - | 2 | -0.39 (-0.93, 0.14)/ M | - | - | - | - | - | - | - | - |

| Study characteristics | Effect Size (95%CI) | Quality of evidence |
| --- | --- | --- |
| Functional outcomes | -0.21 [-0.66, 0.24] | Moderate |
| Muscle strength | -0.19 [-0.63, 0.26] | Moderate |
| Power output | 0.29 [-0.17, 0.76] | Moderate |
| VO2max | 0.39 [-1.12, 1.90] | Very low |
| VO2peak | 0.31 [0.01, 0.61] | High |
| Exercise workload | -10.07 [-34.95, 14.80] | Very low |

**The audience it is intended for**

1. sports scientists who are interested in using hypoxic training,
2. middle-aged and older people who want to improve their health
3. middle-aged and oldery people who have exercise habits.

**Results of Egger’s Test**

VO_2_max


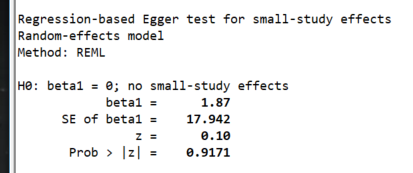


VO_2_peak


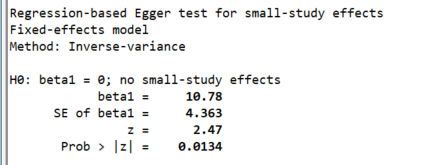


Power output


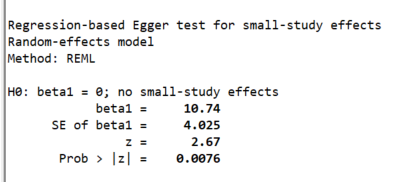


Functional outcome


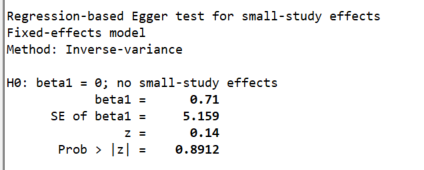


Muscle strength


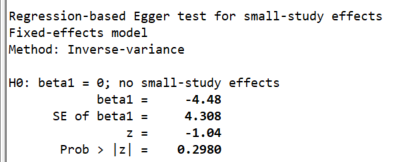


Workload


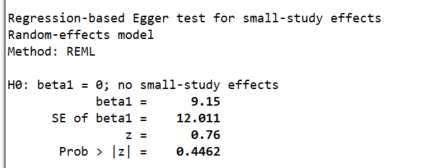

Supplement: Supplemental Information 2 [file peerj-13-19348-s002.docx]
